# Estimating relative abundances of proteins from shotgun proteomics data

**DOI:** 10.1186/1471-2105-13-308

**Published:** 2012-11-19

**Authors:** Sean McIlwain, Michael Mathews, Michael S Bereman, Edwin W Rubel, Michael J MacCoss, William Stafford Noble

**Affiliations:** 1 Department of Genome Sciences, University of Washington, Seattle, WA, USA; 2 Department of Otalaryngology–HNS, Seattle, WA, USA; 3 Department of Physiology & Biophysics, University of Washington, Seattle, WA, USA; 4 Department of Computer Science and Engineering, Seattle, WA, USA

## Abstract

**Background:**

Spectral counting methods provide an easy means of identifying proteins with differing abundances between complex mixtures using shotgun proteomics data. The crux spectral-counts command, implemented as part of the Crux software toolkit, implements four previously reported spectral counting methods, the spectral index (SI_*N*_), the exponentially modified protein abundance index (emPAI), the normalized spectral abundance factor (NSAF), and the distributed normalized spectral abundance factor (dNSAF).

**Results:**

We compared the reproducibility and the linearity relative to each protein’s abundance of the four spectral counting metrics. Our analysis suggests that NSAF yields the most reproducible counts across technical and biological replicates, and both SI_*N *_and NSAF achieve the best linearity.

**Conclusions:**

With the crux spectral-counts command, Crux provides open-source modular methods to analyze mass spectrometry data for identifying and now quantifying peptides and proteins. The C++ source code, compiled binaries, spectra and sequence databases are available at http://noble.gs.washington.edu/proj/crux-spectral-counts.

## Background

Existing methods for differential proteomics (reviewed by [1]) fall into two categories: *spectral counting* methods that rely on counting the number of spectra that map to a given protein across multiple experiments, and *peptide chromatographic peak intensity* methods that use the area under the peptide precursor ion peak as a measure of peptide abundance. In principle, methods based on mass spectrometry peak areas are potentially much more accurate, but these methods require highly reproducible liquid chromatography as well as accurate methods for chromatographic alignment and identification of peaks within the profile spectra. In contrast, spectral counting methods are straightforward to employ and have been shown to correctly detect known differences between samples [2], which contributes to their wide use.

The command line tool crux spectral-counts implements four popular spectral counting methods: the spectral index (SI_*N*_) [3], the exponentially modified protein abundance index (emPAI) [4], the normalized spectral abundance factor (NSAF) [5], and the distributed normalized spectral abundance factor (dNSAF) [6]. The crux spectral-counts command is integrated within the Crux software toolkit, which provides actively maintained open-source methods to identify and now quantify peptides and proteins from shotgun mass spectrometry datasets. Crux supports a variety of input spectra formats, and the tools can easily be incorporated into proteomic analysis pipelines, such as the Trans-Proteomic Pipeline (TPP) [7]. Finally, the modular design of Crux allows improvements to one part of the toolkit to be propagated through downstream analyses.

Currently, several software packages offer spectral counting protein quantification methods [8]. ProteoIQ ( http://www.bioinquire.com) and Scaffold [9] are commercial software products that post-process results from a variety of database search programs. Freely available tools such as APEX [10], emPAI calc [11], and PepC [12] each offer a single spectral counting method. Table 1 compares the features of six software spectral counting tools. Crux offers more spectral counting methods than other tools and is the only method to provide peptide-level in addition to protein-level counts.

**Table 1:** Spectral counting software

	**Crux**	**APEX**	**emPAI Calc**	**PepC**	**ProteoQ**	**Scaffold**
**Metrics**						
**Provided**						
SI_*N*_	X					
emPAI	X		X			
NSAF	X					
dNSAF	X					
Raw	X				X	X
Other		X		X	X	
**Other**						
**Features**						
ParsimonyAnalysis	X				X	X
Peptide-LevelCounting	X					
Free	X	X	X	X		
Opensource	X	X		X		
Web Interface			X	X		
Graphical userinterface		X			X	X
Scriptable	X	X		X		

This table summarizes the features of various spectral counting software methods.

Using crux spectral-counts, we compared and contrasted the reproducibility and linearity of the four spectral counting methods. Our experiments suggest that the NSAF metric provides the most reproducible protein quantification. In contrast, our linearity experiments show that SI_*N*_ and NSAF provide the best performance, with dNSAF providing intermediate performance and emPAI yielding the worst linearity.

The contributions of this paper are thus two-fold: we describe a performance comparison of the reproducibility and linearity of the SI_*N*_, emPAI, NSAF, and dNSAF protein quantification methods, and we provide to the proteomics community a flexible, open source spectral counting software tool.

## Implementation

### Software

The crux spectral-counts command is implemented as part of the Crux proteomics software toolkit [13]. The toolkit is implemented in C++ as a single binary that supports commands for database searching and a variety of downstream analyses [14-18].

The crux spectral-counts command takes as input a protein database in FASTA format and a collection of peptide-spectrum matches (PSMs) produced by a database search procedure. The PSMs may be in Crux’s tab-delimited text format, PeptideProphet’s PepXML or mzIdentML [19]. To compute the SI_*N *_score, a set of spectra must also be provided as input in MS2, mzXML, or mgf format. By default, crux spectral-counts will select the PSMs in the input by a user modifiable threshold of q-value ≤ 0.01.

For each protein with at least one spectral count, the program then computes the NSAF, dNSAF, emPAI, or the SI_*N*_ score. The NSAF metric is defined as

$$\mathrm{NSA}F_{N}=\frac{s_{N}/L_{N}}{\overset{n}{\underset{i=1}{\sum }}(s_{i}/L_{i})}$$

where *N* is the protein index, *s*_*N*_ is the number of spectra matched to protein *N*, *L*_*N *_is the length of protein *N*, and *n* is the total number of proteins in the input database.

The dNSAF metric is given by

$$\text{dNSA}F_{N}=\frac{\frac{s_{N}^{u}+\overset{k}{\underset{j=1}{\sum }}d_{j,N}s_{j,N}^{s}}{L_{N}}}{\overset{n}{\underset{i=1}{\sum }}\frac{s_{i}^{u}+\overset{k}{\underset{j=1}{\sum }}d_{j,i}s_{j,i}^{s}}{L_{i}}}$$

where $s_{n}^{u}$ is the spectral count for the peptides uniquely mapping to protein *N*, $s_{j,N}^{s}$ is the spectral count of degenerate peptide *j* (out of the protein’s *k* degenerate peptides) mapped to protein *N*, and *d*_*j*,*N*_ is the distribution factor of peptide shared counts, defined by the equation

$$d_{j,N}=\frac{s_{N}^{u}}{\overset{n}{\underset{i=1}{\sum }}s_{i}^{u}}$$

The metric emPAI is defined as

$$\mathrm{emPA}I_{N}=\frac{\left(10^{\frac{p_{N}^{\text{observed}}}{p_{N}^{\text{observable}}}}-1\right)}{\overset{n}{\underset{i=1}{\sum }}\left(10^{\frac{p_{i}^{\text{observed}}}{p_{i}^{\text{observable}}}}-1\right)}$$

where $p_{N}^{\text{observable}}$ is the number of unique peptides observable for protein *N* and $p_{N}^{\text{observed}}$ is the number of unique peptides observed for protein *N*.

Finally, the SI_*N*_ score is calculated using

$$SI_{N}=\frac{\overset{p_{N}}{\underset{j=1}{\sum }}\left(\overset{s_{j}}{\underset{k=1}{\sum }}i_{k}\right)}{L_{N}\left(\overset{n}{\underset{j=1}{\sum }}SI_{j}\right)}$$

where *p*_*N*_ is number of unique peptides in protein *N*, *s*_*j*_ is the number of spectra assigned to peptide *j*, and *i*_*k *_is the total fragment ion intensity of spectrum *k*. Analogous scores can also be computed for each peptide, rather than for each protein. A detailed description of the peptide-level scoring metrics is available in the on-line documentation. As output, crux spectral-counts produces a tab-delimited file listing proteins and their corresponding counts, in reverse sorted order.

The crux spectral-counts command also computes a parsimonious set of proteins, using the greedy set cover approach used by IDPicker [20]. Users thus have the option of considering spectral counts only for proteins within the parsimonious set.

### Data Collection

For the reproducibility experiments, proteins were extracted from the cochlear nucleus of the developing mouse brain at postnatal day 7 and postnatal day 21. Two biological replicates were generated for each age by dissecting out the cochlear nuclei from two separate mice at each age. One of the 21-day mice was used to generate two samples, thereby providing a technical replicate in addition to a biological replicate. The samples prepared from the chicken brain were derived from nucleus laminaris, an auditory region in the brain stem. Samples were taken from the dorsal (D) and ventral (V) regions of this area. For each region, two biological replicates were generated, and one of those replicates was also subjected to technical replication. Each sample was digested with trypsin and subjected to liquid chromatography followed by tandem mass spectrometry.

For the linearity experiments, we used eight samples that represent a dilution curve of 48 known proteins synthesized by Sigma (UPS1, http://www.sigmaaldrich.com). These data sets are mixtures (Std1–Std8) of the *C. elegans* lysate at equal concentrations and the 48 proteins, diluted by a two-fold in each successive standard. Std 8 has the lowest concentration of the known proteins (6 fmol) and Std 1 has the highest concentration (870 fmol).

All three data sets are publicly available at http://noble.gs.washington.edu/proj/crux-spectral-counts.

### Data analysis

The fragmentation spectra from the experiments were searched against their respective mouse, chicken, or the C. elegans+UPS1 protein database using crux sequest-search followed by crux q-ranker, with the default parameters. crux spectral-counts was applied to the peptide-spectrum matches (PSMs) that received *q*-values ≤ 0.01. The resulting data sets for the mouse and chicken replicates are summarized in Additional file 1: Table S1, and the UPS1 dilution curve data sets are summarized in Additional file 1: Table S2.

## Results

### Testing reproducibility between replicates

**Figure 1:**
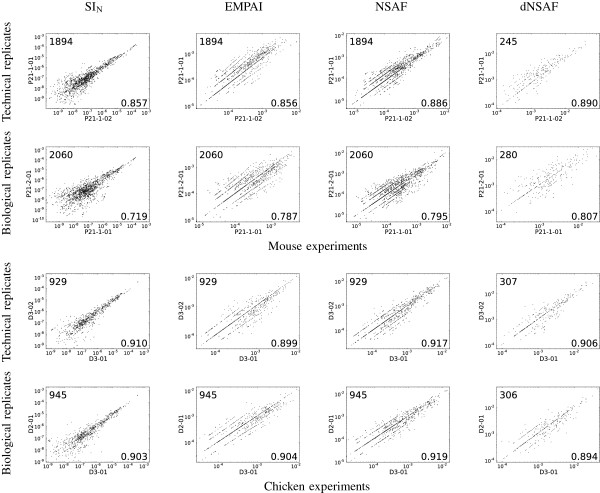
**Reproducibility of spectral counts across biological and technical replicate experiments.** Each plot compares either the SI_*N*_, emPAI, NSAF or dNSAF measure for proteins that were reproducibly identified across two replicate experiments. For visualization purposes, the counts are plotted on a logarithmic scale. The number in the lower right corner of each panel is the corresponding Spearman correlation and the number in the upper left is the number of datapoints compared.

To investigate the reproducibility of the four spectral count methods, we analyzed mass spectrometry data from technical and biological replicates from chicken and mouse samples. We then produced a scatter plot for each pair of biological or technical replicates and computed the corresponding Spearman correlation. For these comparisons, proteins identified in only one of the two datasets were ignored. Figure 1 shows sixteen such plots, corresponding to one biological and one technical replicate for chicken and mouse, respectively. The complete collection of 76 plots is provided as Additional file 1: Figures S1–S2. From these analyses, as summarized in Table 2, we draw two primary conclusions. First, the spectral counts are generally reproducible: the mean correlation value across all 76 pairs is 0.867, and the minimum correlation is 0.719. Second, reassuringly, the technical replicates produce higher correlations than the biological replicates: the mean correlation among 24 pairs of technical replicates is 0.885, whereas the corresponding value for the 52 pairs of biological replicates is 0.859 (two-tailed Wilcoxon rank-sum test p-value=0.026).

**Table 2:** Spectral-counting reproducibility performance on mouse and chicken replicates

**Metric**	**Technical**	**Biological**	**All Replicates**
SI_*N*_	0.885	0.848	0.859
emPAI	0.870	0.858	0.862
NSAF	0.899	0.876	0.884
dNSAF	0.886	0.852	0.863
All Metrics	0.885	0.859	0.867

This table summarizes the average correlation of the spectral-counting metrics across the technical and biological replicates.

**Figure 2:**
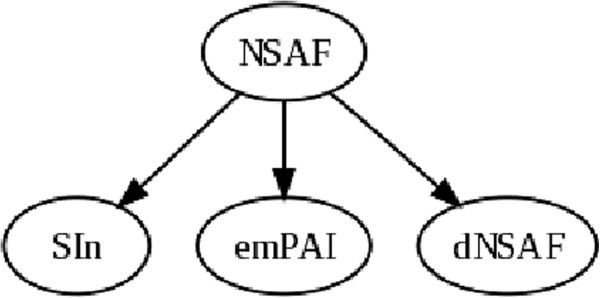
**Comparison of spectral counts across replicates.** This graph summarizes the statistical analysis of the reproducibility measurements. An edge leading out from node *A* to node *B* indicates a statistically significant improvement in reproducibility for method *A* relative to method *B*.

To test whether the observed differences in correlations among the four metrics are significant, we applied a Wilcoxon signed-rank test to paired sets of correlations. With four metrics, there are six possible paired comparisons. Figure 2 shows the results of this analysis, where one metric attaining a significant increase (using a Bonferroni p-value of 0.05/6=0.008333) over another is indicated by a directed edge. From this graph we conclude that, for the biological and technical replicates, NSAF yields significantly more reproducible quantification values than SI_*N*_, dNSAF and emPAI. Our reproducibility results agree with Colaert et al., who claim that NSAF is more reproducible than SI_*N *_and emPAI [21]. However, in contrast to our results, Griffen et al. report better reproducibility across replicates for SI_*N *_compared to NSAF [3].

### Testing linear response for protein abundance across samples

**Figure 3:**
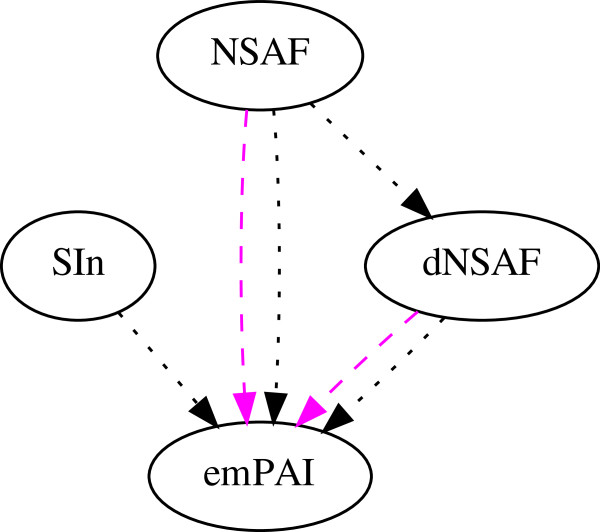
**Comparison of spectral counts across UPS1 dilution curve.** This graph summarizes the statistical analysis of the linearity measurements. Two types of analysis were performed, using the linear regression correlation, *R*^2 ^and mean percent error (MPE) for the *C. elegans* + UPS1 dilution curve dataset. An edge leading out from node *A* to node *B* indicates a statistically significant improvement in linearity for method *A* relative to method *B*.

To determine the linear response of each of the spectral count metrics, we analyzed mass spectra from a dataset of samples that form a dilution curve of forty-eight proteins with known amounts spiked into a *C. elegans* lysate. We performed linear regression between each protein spectral count and the associated amounts across the dilution curve samples. For this analysis, we only included proteins that obtain a positive spectral count in three or more of the data sets, which results in a comparison of forty-two proteins across the four metrics. We then carried out a Wilcoxon signed rank test analysis separately on the average correlation, *R*^2^, and the mean percent error (MPE). The results of these tests (Figure 3) are fairly consistent with one another: NSAF significantly outperforms dNSAF, and dNSAF and SI_*N*_significantly outperform emPAI.

Colaert et al. (2011) claim that SI_*N *_is more accurate than both NSAF and emPAI [21], but we find evidence only to support the former claim, even though our experiments employ a wider dynamic range of protein abundance (6.7–20 fmol versus 6–870 fmol) and more data sets (two versus eight). Based on our experiments, we conclude that NSAF or SI_*N*_ are the methods of choice for ensuring an accurate linear response between a protein’s change in abundance across different samples.

It is worth noting that Griffin et al. (2010) observe a good linear fit between SI_*N *_and protein quantification. However, their evaluation methodology fits a single line to all of the SI_*N*_ values from many proteins, whereas we have fit a separate line for each protein. This difference reflects our belief that spectral counting methods are most useful as measures of the relative abundance of a single protein between two experiments. We did not test the claim that SI_*N *_provides an accurate absolute protein abundance metric.

## Discussion

Overall, our experiments suggest a relative ordering of spectral counting methods according to their reproducibility and the linearity of their response, but we can only speculate as to the reasons for the ranking that we observe. For example, we note that NSAF outperforms the emPAI metric in both of our experiments. The emPAI measure takes into account the least information—not only does it ignore fragment ion intensities, but emPAI also fails to account for the length of the protein. Apparently, this relatively simple approach is insufficient to accurately estimate protein abundance. The relative performance of NSAF and SI_*N*_, on the other hand, is less clear: NSAF yields more reproducible results than SI_*N*_ but the two methods are statistically indistinguishable with respect to linearity. The main difference between SI_*N*_ and the other three metrics is that SI_*N *_is the only metric that takes into account the intensities of the fragment ion peaks. In this sense, SI_*N*_ goes a bit beyond the strict definition of “spectral counting.” Our experiments do not support the claim that such intensity information is valuable for quantification. However, the conflicting results of our study and Collaert et al., on the one hand, versus Griffin et al. on the other hand, suggests perhaps that further comparison of these methods is warranted.

An additional direction for future work involves quantifying the linearity and reproducibility of proteins in a segregated fashion according to protein abundance. For example, visual inspection of Figure 1 suggests that perhaps the SI_*N *_measure yields more reproducible counts for high abundance proteins, with a corresponding decrease in reproducibility as the abundance drops. Arguably, in many studies, such low abundance proteins are of the greatest interest; hence, it may be worthwhile to investigate in a systematic fashion the extent to which either the linearity or the reproducibility of a given spectral counting measure varies as a function of protein abundance.

## Conclusions

Quantifying protein amounts in mass spectrometry by spectral counting is a simple and robust method for measuring the relative change of protein amounts across different samples; however, many different algorithms exist for assigning a score to each identified protein. Using crux spectral-counts, we compared and contrasted four spectral counting methods with respect to their reproducibility across replicates and their linear response relative to protein abundance. Crux provides a flexible, easy to use open source tool for performing protein quantification using spectral counting.

## Availability and requirements

**Project name:** Crux tandem mass spectrometry analysis software

**Project home page:**http://noble.gs.washington.edu/proj/crux

**Operating systems:** Linux, MacOS, Windows + Cygwin

**Programming language:** C++

**Other requirements:** Crux has no requirements to install the binary version under Linux or MacOS. On Windows, Crux requires Cygwin. To compile Crux requires a c++ compiler, cmake, and Subversion.

**License:** Apache

**Any restrictions to use by non-academics:** None

## Competing interests

The authors declare that they have no competing interests.

## Authors’ contributions

The chicken and mouse samples were provided by ER’s lab, and the LC-MS/MS data were collected by members of the MM lab. MB prepared and collected the UPS1 + *C. elegans* dilution sample datasets. MM wrote the initial code for crux spectral-counts and the initial draft of the manuscript. SM finished the coding of crux spectral-counts and the final draft with WSN. All authors revised and approved the final manuscript.

## Supplementary Material

Additional file 1 **Supplementary Information.** Supplementary Tables 1 and 2 and Suplementary Figures 1 and 2 are provided as quantify-supplement.pdf. Click here for file
