# Supplementary material for: Estimating relative abundances of proteins from shotgun proteomics data
Source: BMC Bioinformatics. 2012 Nov 19;13:308. doi: 10.1186/1471-2105-13-308 (PMC3599300; doi:10.1186/1471-2105-13-308)
Supplement: Additional file 1 — Supplementary Information. Supplementary Tables 1 and 2 and Suplementary Figures 1 and 2 are provided as quantify-supplement.pdf. [file 1471-2105-13-308-S1.pdf]

Supplement to “Crux spectral-count: estimating relative abundances of proteins from shotgun proteomics data”

Sean McIlwain[1] , Michael Mathews[1] , Michael S. Bereman[1] , Edwin W. Rubel[2,3] , Michael J. MacCoss[1] , William Stafford Noble*[1,4]

[1]Department of Genome Sciences, University of Washington, Seattle, WA, USA
[2]Department of Otalaryngology–HNS, University of Washington, Seattle, WA, USA
[3]Department of Physiology & Biophysics, University of Washington, Seattle, WA, USA
[4]Department of Computer Science and Engineering, University of Washington, Seattle, WA, USA

Email: William Stafford Noble - william-noble@uw.edu;

*Corresponding author

Supplementary Table 1- Mouse and Chicken proteomics data sets

The first column indicates the species from which the sample was taken. The experiment identifier indicates the postnatal day for mouse samples and brain region for chicken samples, the biological replicate number and the technical replicate number. The third and fourth columns indicate the total number of spectra collected, and the number of peptide-spectrum matches (PSMs) with $q \leq 0.01$.

Species	Experiment	spectra	PSMs
mouse	P7-1-01	21819	5464
mouse	P7-2-01	18969	5115
mouse	P21-1-01	20059	5142
mouse	P21-1-02	21289	4265
mouse	P21-2-01	18943	5196
mouse	P21-2-02	21819	4722
chicken	D2-01	17657	3085
chicken	D3-01	18538	3332
chicken	D3-02	19149	3199
chicken	NL-1-01	19073	3516
chicken	NL-1-02	19640	3578
chicken	NL-2-01	20351	4070
chicken	NL-2-02	20748	4334
chicken	V1-01	18365	3702
chicken	V3-01	18516	3821
chicken	V3-02	16684	3168

Supplementary Figure 1- Biological and technical replicate correlations of mouse samples using spectral counts

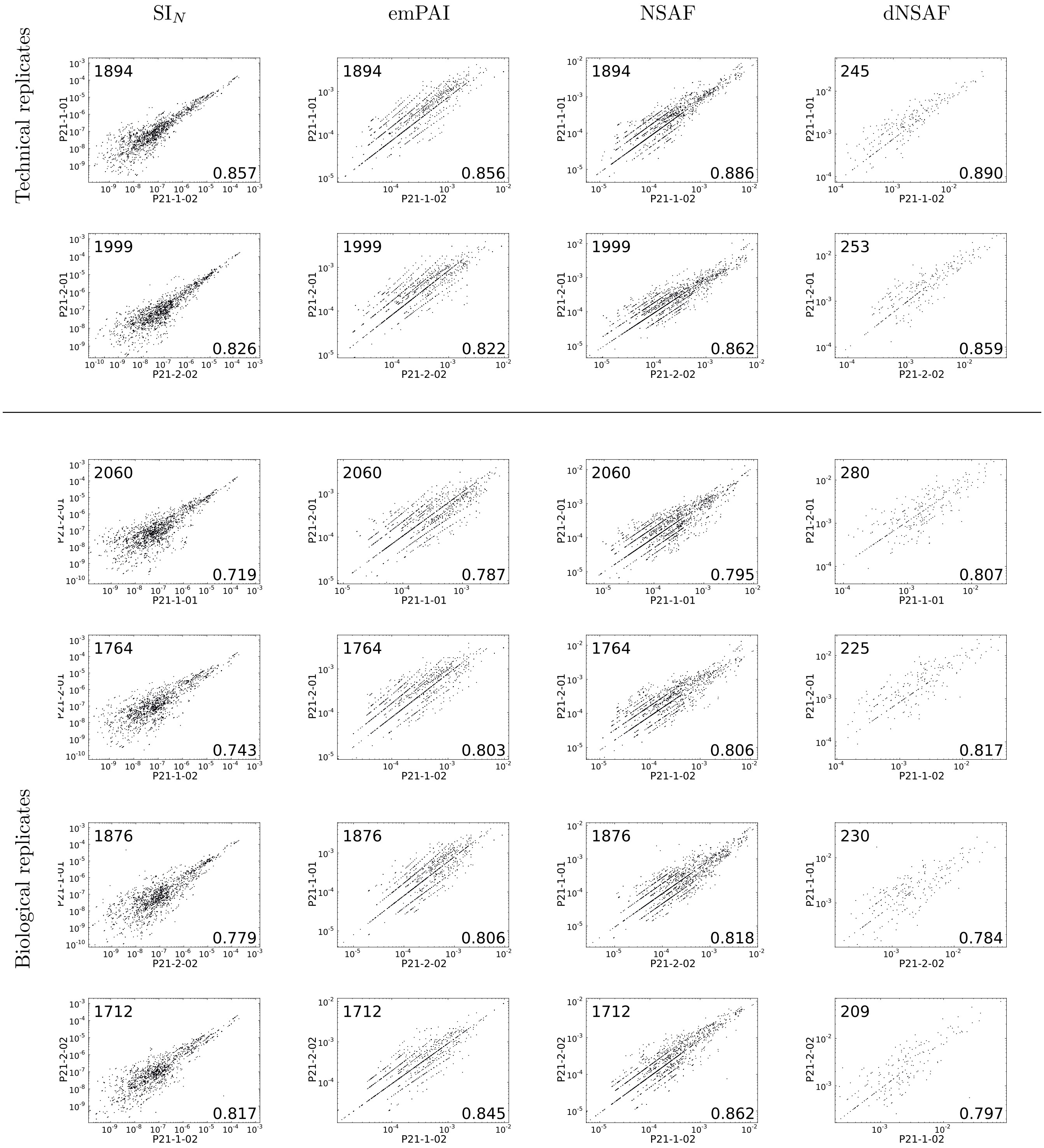

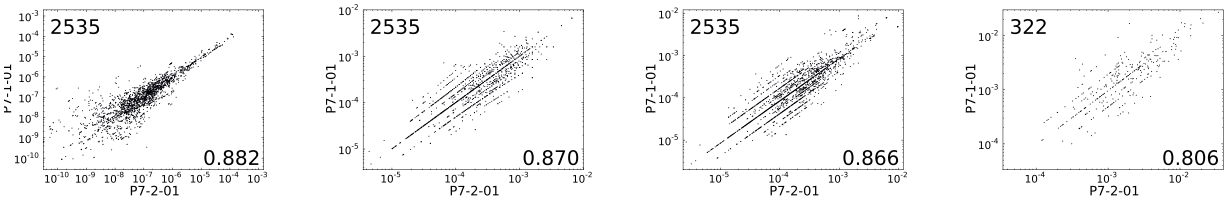
2535
0.882
2535
0.870
2535
0.866
322
0.806
P7-2-01
P7-1-01

Supplementary Table 2 - Biological and technical replicate correlations of chicken samples using spectral counts

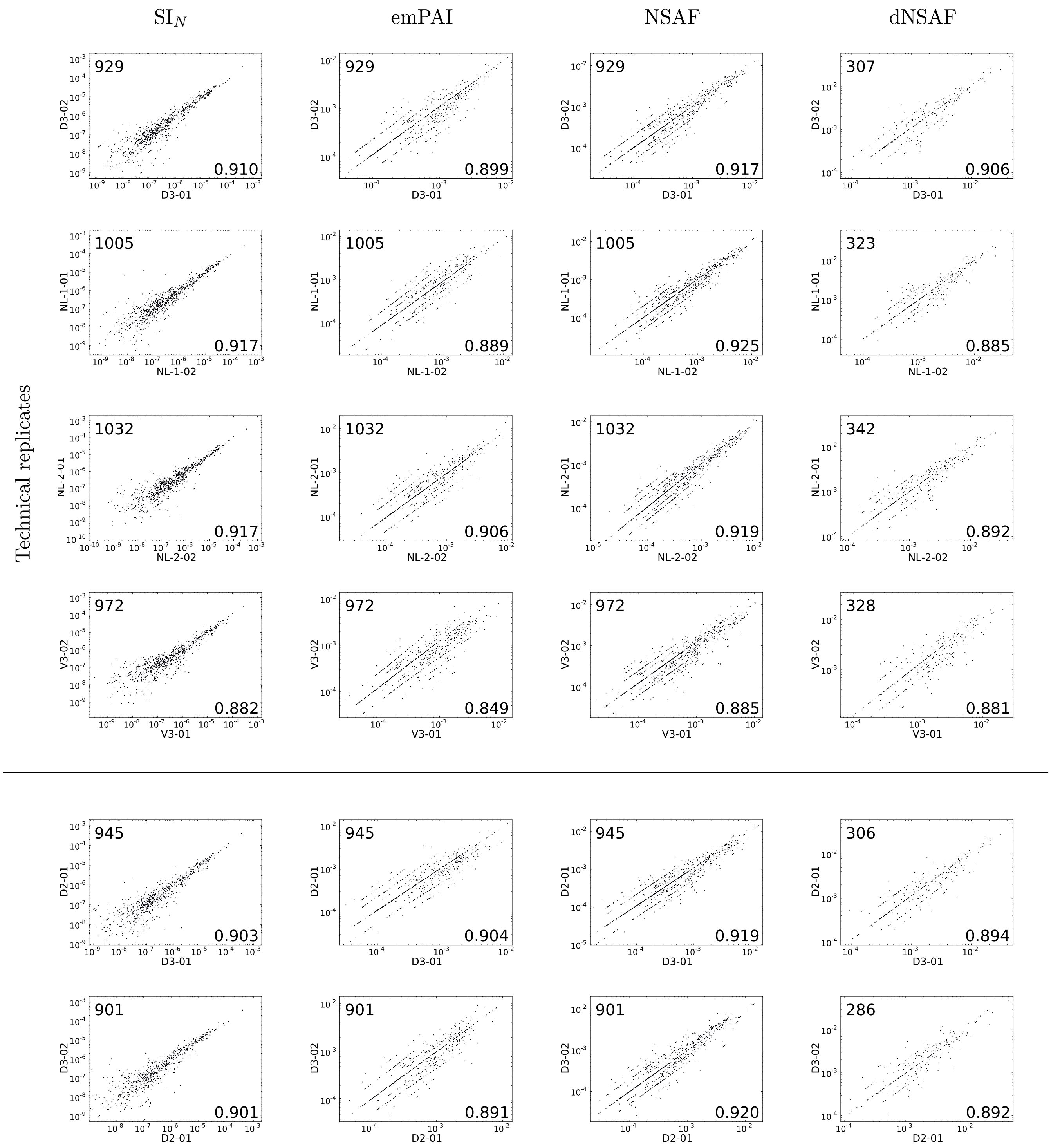

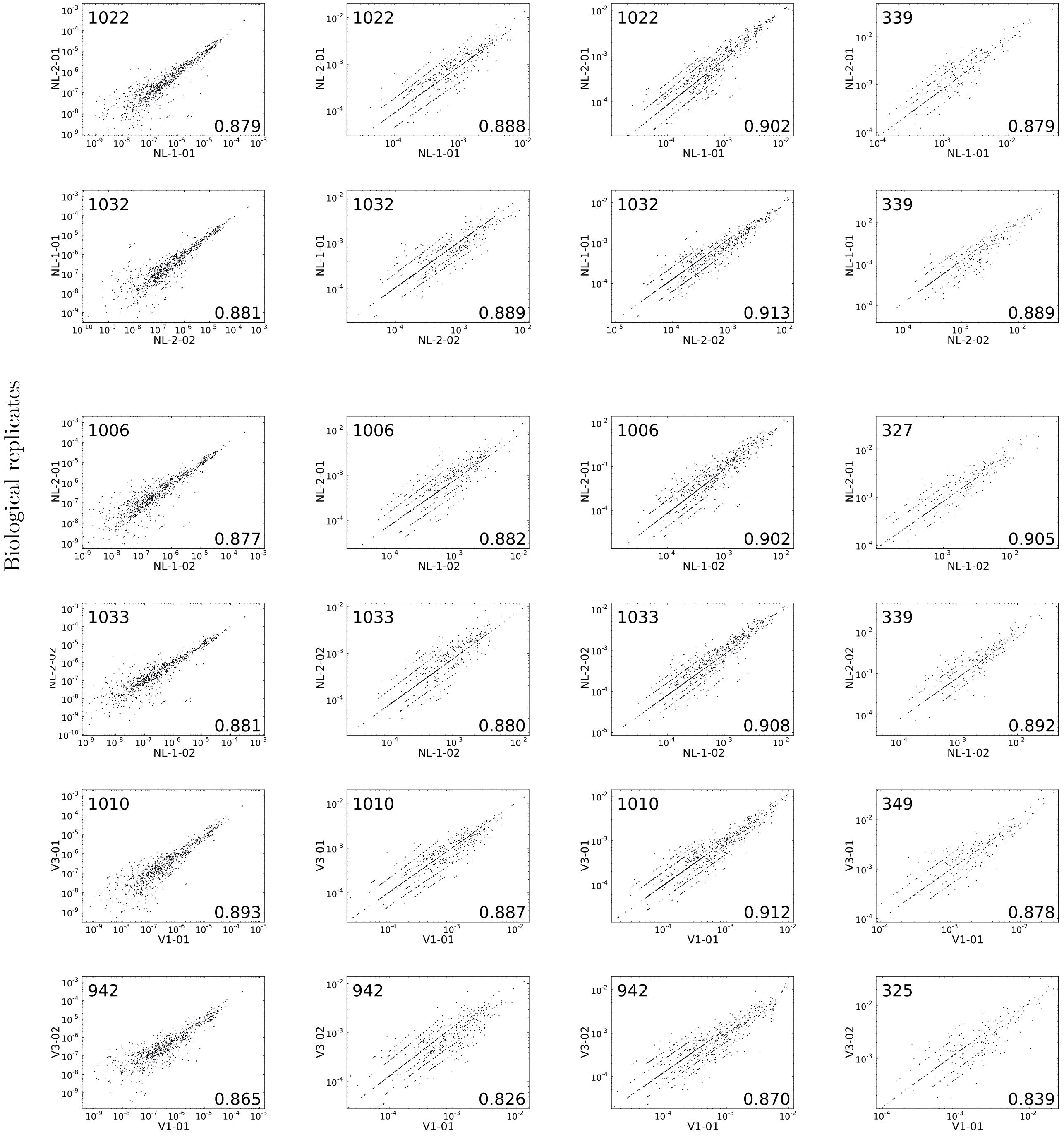

Biological replicates
NL-2-01
1022
0.879
1022
0.888
1022
0.902
339
0.879
NL-1-01
NL-1-01
1032
0.881
1032
0.889
1032
0.913
339
0.889
NL-2-02
NL-2-01
1006
0.877
1006
0.882
1006
0.902
327
0.905
NL-1-02
NL-2-02
1033
0.881
1033
0.880
1033
0.908
339
0.892
NL-1-02
V3-01
1010
0.893
1010
0.887
1010
0.912
349
0.878
V1-01
V3-02
942
0.865
942
0.826
942
0.870
325
0.839
V1-01

Supplementary Table 2 - UPS1 Shotgun proteomics datasets

The first column indicates the species from which the sample was taken. The experiment identifier indicates the postnatal day for mouse samples and brain region for chicken samples, the biological replicate number and the technical replicate number. The third and fourth columns indicate the total number of spectra collected, and the number of peptide-spectrum matches (PSMs) with $q \leq 0.01$.

Experiment	Amount UPS1 on Column (fmol)	spectra	PSMs
Std 1	870	24758	6110
Std 2	435	25882	8261
Std 3	217	25660	9203
Std 4	109	25125	9387
Std 5	54	25117	9675
Std 6	27	25603	9654
Std 7	13	25451	9664
Std 8	6	25653	9656
